# Learned Doctors

**Published:** 1871-01

**Authors:** 


### LEARNED DOCTORS.

The American Medical Association held a Convention recently at Washington city. Among the subjects discussed was, whether young men attending medical lectures with a view to become physicians should be required to have some knowledge of Latin and Greek. The decision was, in effect, that a man may graduate and obtain a degree of M. D. without having studied the languages above named. We are very much afraid that what with quackery, charlatanism, impudence, brass, and bought diplomas, the medical profession is going, or has gone, to the dogs in this country.

We regret, for the sake of his honored father, our teacher in the Medical Department of Transylvania University, then only second in numbers, and second in ability to no medical institution on this continent, that Prof. W. D. Yandell was opposed practically to a high literary requisition for medical students. That a man may become eminent both in medicine and surgery without a knowledge of either Greek or Latin is not denied; but that the lowering the standard of learning in any of the learned professions, must inevitably degrade that profession, cannot for a moment be doubted. That a man should commit his life to the hands of an ignoramus is terrible to think of. The Association would have done better to have required a diploma from the first colleges in the land as an indispensible prerequisite to admission to a medical college.

